# The nitrate–nitrite–nitric oxide pathway of neuroinflammation and cognitive impairment in ischemic stroke

**DOI:** 10.3389/fimmu.2026.1790943

**Published:** 2026-03-23

**Authors:** Jinyan Liu, Qiuyue Deng, Shufei Wei, Lin Cheng, Chunxiao Shen, Ziying Liu, Yuemin Qiu, Jiahui Teng, Liangliang Wang, Xiaorong Zhang

**Affiliations:** 1 Department of Pathology, Affiliated Hospital of Jiujiang University, Jiujiang, Jiangxi, China; 2 Jiujiang Clinical Precision Medicine Research Center, Jiujiang, Jiangxi, China; 3 Department of Neurology, Affiliated Hospital of Jiujiang University, Jiujiang, Jiangxi, China

**Keywords:** ischemic stroke, neuroinflammation, neuroprotection, nitrate-nitrite pathway, nitric oxide

## Abstract

Ischemic stroke is the most prevalent type of stroke worldwide and poses a serious threat to human health. Neuroinflammation following an ischemic stroke is a key factor in cognitive impairment and disease progression, yet effective interventions are lacking. The classical L-arginine-nitric oxide synthase (L-arginine-NO) pathway for nitric oxide (NO) synthesis becomes impaired under ischemic and hypoxic conditions. This review focuses on an essential alternative pathway: the nitrate-nitrite-NO pathway, which is preferentially activated under hypoxic conditions and serves as a ‘backup system’ for maintaining NO bioavailability. This review outlines the epidemiology and pathomechanisms of ischemic stroke and introduces the biochemical basis of the NO pathway. It emphasizes the pathway’s dual role in ischemic stroke, exerting anti-inflammatory and neuroprotective effects by mediating vasodilation, improving cerebral perfusion, regulating microglial polarization, preserving blood–brain barrier integrity, and promoting synaptic plasticity. This mitigates cognitive impairment. Conversely, the review also explores the potential neurotoxic effects of excessive NO during the late reperfusion phase. Furthermore, this review discusses novel therapeutic strategies based on this pathway, including exogenous NO supplementation (e.g., dietary nitrates or NO donor drugs), gene therapy, targeted delivery systems incorporating nanotechnology, and combination therapies with other medications. These strategies are all designed to enhance treatment precision and bioavailability. Finally, we summarize current research limitations and highlight potential directions for future investigations. We conclude that an in-depth exploration of the nitrate-nitrite-NO pathway provides strong theoretical foundations and novel perspectives for developing innovative treatments targeting neuroinflammation and cognitive impairment after ischemic stroke.

## Graphical Abstract

**Figure:**
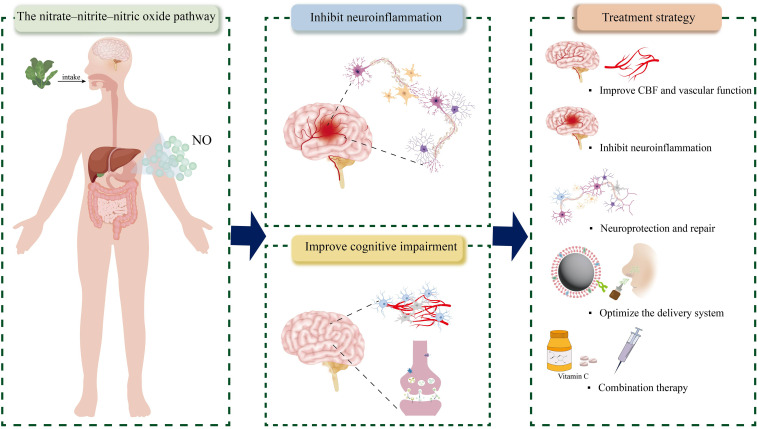

## 1 Introduction

Ischemic stroke is one of the world’s most significant health issues, which can be described as an interruption of cerebral blood supply caused by occlusion or severe narrowing of cerebral blood vessels. This reduces cerebral circulation, leading to ischemia and hypoxia, which results in the death of brain cells in the affected vascular supply area (1). As per research, stroke incidence is on the rise, and it has been estimated that one in four people worldwide has suffered a stroke (2). Symptoms of stroke may be sudden weakness or numbness in a leg or arm, facial paralysis, difficulty speaking or understanding speech, confusion, difficulty with coordination or balance, and vision changes (3), Neuroinflammation is an essential pathological process (4–6)As growing understanding of neuroinflammation-induced cognitive impairment in ischemic stroke develops, promoting and inhibiting its progression increases ever greater significance.

Experimental evidence (7, 8) indicates that the nitrate-nitrite-nitric oxide (nitrate-nitrite-NO) pathway promotes the discharge of nitric oxide (NO) under acidic and/or hypoxic environments, replacing the traditional L-arginine-dependent NO production pathway as a significant source (9). Under such circumstances, nitric oxide synthase (NOS)-independent NO production can be viewed as a reservoir that preserves adequate NO production under decreased oxygen availability (10).

The exogenous NO pathway has not been the subject of intense investigation in stroke research to date. However, this pathway can cause considerable dilation of blood vessels, thereby increasing blood flow in cases of ischemia, hypoxia, and disease. During physiological and pathological hypoxia, the production of NO and NO-modified proteins from nitrite reduction has been implicated in physiological hypoxic signaling, vasodilation, reduction in blood pressure, regulation of cell respiration, regulation of oxidative stress, and cellular protection against ischemia/reperfusion injury (7, 11–13). This actually prevents complications such as ischemic stroke from leading to pathological conditions.

This article essentially addresses the pathogenesis of ischemic stroke and the therapeutic strategy based on exogenous NO. The article specifies the role of the nitrate-nitrite-NO pathway in ischemic stroke, as well as its impact on neuroinflammation and cognitive impairment in the condition. The article specifically addresses the clinical application of NO in ischemic stroke, aiming to present evidence-based management for its treatment.

## 2 Overview of ischemic stroke

Stroke is a neurological illness that clinically manifests as an acute or transient deficit of cerebral function. Stroke is among the three most common diseases in the world (14), with a high rate of mortality and disability, and a catastrophic impact on the health of humans and on the quality of life. Strokes are categorized into ischemic or hemorrhagic strokes. Ischemic stroke occurs due to decreased cerebral blood flow (CBF) to an area of the brain due to vascular occlusion (15), leading to necrosis of brain tissue and localized loss of neurons (16). On the other hand, hemorrhagic stroke occurs when leaking cerebral vessels cause hemorrhage within the brain or subarachnoid space (17–19). Ischemic stroke is the most prevalent and accounts for 87% of all strokes. The ischemic stroke cascade (20) includes neuroinflammation, disruption of the blood-brain barrier (BBB), and ultimately cell death. This article primarily addresses ischemic stroke.

At the molecular level, ischemic stroke is caused by reduced cerebral perfusion, leading to oxygen and glucose deprivation. The deprivation subsequently impairs adenosine triphosphate (ATP) synthesis, leading to lactic acidosis and cellular disruption of homeostasis (16). Lactic acidosis causes cellular injury by disrupting normal brain acid-base homeostasis. Additionally, ATP deficiency disrupts the function of ATP-dependent ion-transporting pumps, resulting in cell depolarization and the opening of voltage-gated Ca²^+^, Na^+^, and K^+^ channels (16). This leads to bulk Ca²^+^and Na^+^ entry in association with K^+^ efflux, collectively releasing glutamate and resulting in glutamate-mediated extracellular excitotoxicity (21). Concurrently, increased intracellular Na^+^ entry causes cytotoxic edema (21), and elevated Ca²^+^ levels activate proteases and lipases, triggering the release of free radicals and destroying crucial cellular components, such as mitochondria. Concurrent with excitotoxicity, hypoxia- and reperfusion-evoked neuroinflammation is another significant contributor to stroke pathophysiology. Furthermore, more and more evidence indicates (22, 23) that post-ischemic inflammation leads to secondary development of brain injury, and the severity of stroke outcome in comorbidity is dependent on the degree of inflammation.

### 2.1 Neuroinflammation in ischemic stroke

Ischemic stroke and neuronal injury studies reveal that neuronal injury is caused by neuron loss, oxidative stress, and immune reaction (24, 25). Neuroinflammation is the activation of the brain’s intrinsic immune system in response to a stimulus, such as cerebral ischemia, which recruits immune cells, the cerebral vasculature, and molecular mediators (26, 27). Neuroinflammation is increasingly accepted as an essential element of stroke pathophysiology that impacts both acute and chronic stages of the condition. In Acute Ischemic Stroke (AIS) (26, 28), neuroinflammation is initiated within minutes of ischemia onset. It lasts for days, involving the activation of resident immune cells, including microglia and astrocytes, and the recruitment of peripheral immune cells (20). It triggers the production of proinflammatory cytokines, chemokines such as CXCL8, CCL2, and CCL3, and reactive oxygen species (ROS). These inflammatory mediators contribute to the disruption of the blood-brain barrier. Cerebral edema and neuronal injury induce neuronal apoptosis and disrupt neural plasticity, which, in turn, worsen neurological deficits. The inflammatory cascade amplifies the initial brain injury, resulting in secondary injury, preventing tissue repair and recovery of function, and rendering the patients vulnerable to post-stroke complications, including motor and cognitive impairment.

#### 2.1.1 Role of brain cells in ischemic stroke

##### 2.1.1.1 Microglia

Microglia, the predominant macrophage-like cells resident in the central nervous system (CNS), are the key defense against stroke. Microglia, activated by ischemia, exhibit both neurotoxic and neuroprotective effects (29). During cerebral ischemia, microglia rapidly activate and release pro-inflammatory cytokines, including Tumor Necrosis Factor-α (TNF-α) and Interleukin-β (IL-β), which amplify inflammation (30, 31). Aside from their role in inflammatory mechanisms, microglia also contribute to maintaining healthy neural circuits during development and repair, as well as to removing disease-causing proteins (4). Microglia enable the unimpeded passage of small molecules and charged ions via gap junctions, which are special channels. Microglia and other neurons also support synaptic maintenance and function, thereby preventing cognitive impairments associated with synaptic dysfunction (32). For instance, post-ischemic inflammation is controlled through microglial P2X4 receptors (33), while the absence of P2X4R blocks acute stroke but induces chronic susceptibility to depression-like behavior following stroke. Therefore, time-dependent approaches must be employed when targeting P2X4R after stroke (34). In contrast, during the recovery phase, activation of P2X4 receptors (P2X4Rs) on microglia promotes the release of brain-derived neurotrophic factor (BDNF), which relieves depressive symptoms, preserves synaptic plasticity, and accelerates behavioral recovery after stroke.

##### 2.1.1.2 Astrocytes

During ischemia, impaired expression of excitatory amino acid transporter 2 (EAAT2), a glutamate transporter predominantly localized in astrocytes, compromises astrocytic glutamate uptake and leads to excitotoxicity and neuronal death (35). However, astrocytic glutamate uptake is very energy-demanding, and this poses a problem in ischemic stroke. Moreover, under ischemic conditions, astrocytes proliferate and become activated in the presence of cytokines, releasing vimentin, IL-1β, chemokine protein-1, and MMPs, which further destabilize the blood-brain barrier (36, 37). Specific solutions for addressing the high energy cost of glutamate uptake will be discussed in the following sections.

### 2.2 Cognitive impairment due to ischemic stroke

Cognitive impairment is a common sequelae of stroke, significantly impacting patients’ quality of life. Post-stroke cognitive impairment (PSCI) is defined as any degree of cognitive impairment, ranging from mild cognitive impairment to dementia, that occurs after a clinically evident stroke (18). Its diagnosis is primarily based on temporal association, meaning the cognitive decline must occur following a stroke event (38, 39).

It is crucial to distinguish PSCI from vascular cognitive impairment (VCI) and vascular dementia (VaD). VCI is a broader umbrella term encompassing all forms of cognitive impairment caused by cerebrovascular disease and its risk factors, with or without a history of overt stroke (32). Its etiology includes not only multiple infarcts but also cerebral small vessel disease (e.g., leukoaraiosis, lacunes), intracerebral hemorrhage, and mixed pathologies (32). VaD represents the most severe stage of the VCI spectrum, meeting the diagnostic criteria for dementia.

In essence, PSCI emphasizes the stroke event as the trigger for cognitive decline, whereas VCI/VaD emphasizes the vascular etiology. While most cognitive deficits resulting from a clear stroke fall under the VCI umbrella, individuals with “silent” cerebral small vessel disease can develop VCI without ever experiencing a clinical stroke, thus not meeting the definition of PSCI. Therefore, a clear distinction based on etiology and clinical context is essential in research.

In addition to the main effect of stroke, post-stroke cognition is also influenced by other stroke complications (e.g., delirium, hyponatremia, depression), pre-stroke cognitive impairment, and comorbid age-related neuropathology (40).

## 3 Nitrate-nitrite-NO pathway

Inorganic anions nitrate (NO_3_^-^) and nitrite (NO_2_^-^) were traditionally considered inert end products of endogenous NO metabolism. However, it appears that these anions cyclically interconvert to regenerate NO *in vivo*, selectively releasing NO under conditions of acid and/or hypoxia. This pathway is a vital alternative to the classic L-arginine-based synthesis pathway for NO generation. Because the L-arginine-NOS pathway is oxygen-dependent, the nitrate-nitrite-NO pathway becomes increasingly active with decreasing oxygen tension. Therefore, NOS-independent NO production may be regarded as a safety mechanism that guarantees adequate NO generation under circumstances of restricted oxygen levels—comparable to the compensatory role of anaerobic glycolysis in energy homeostasis. This idea enforces the proposal that nitrite is involved in hypoxic vasodilation and modulates mitochondrial oxygen use. It also suggests an intriguing possibility that nitrite is involved in cytoprotective signaling during pathological ischemia and reperfusion (41).

**Figure 1:**
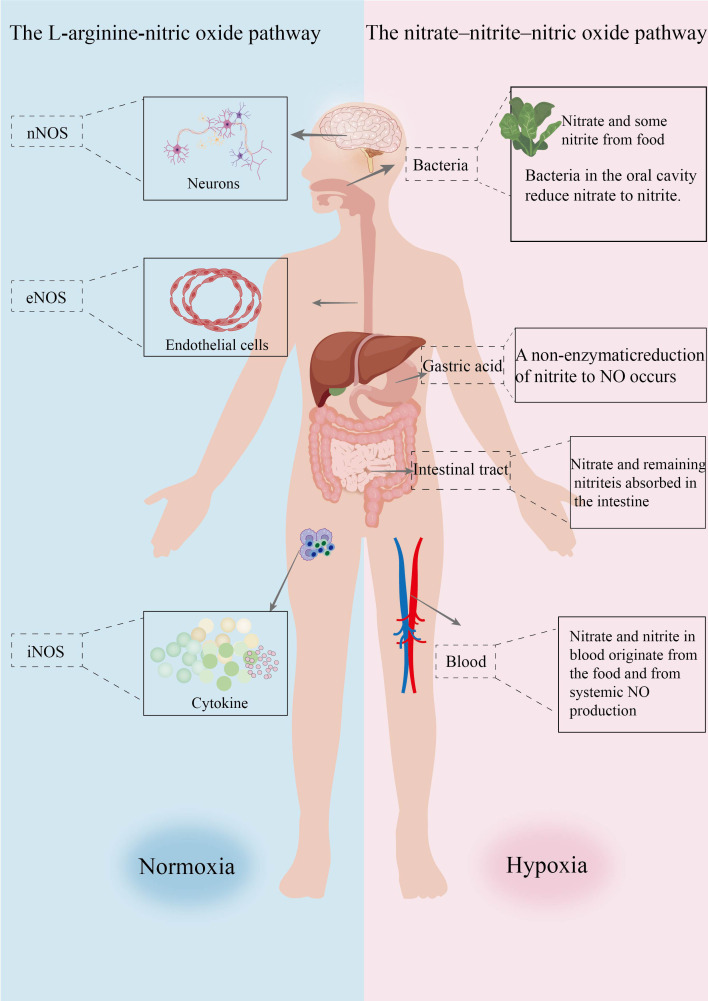
Endogenous NO pathways and exogenous NO pathways. (This diagram illustrates two pathways for NO production within the body. The left section (normoxic environment) depicts the L-arginine-NO pathway, in which nNOS participates in NO generation within neurons, eNOS within endothelial cells, and iNOS is induced by cytokines. The right-hand section (hypoxic environment) depicts the nitrate-nitrite-NO pathway. Nitrates and some nitrites from dietary sources are reduced to nitrites by oral bacteria, then converted to NO via non-enzymatic reduction by gastric acid in the stomach. The remaining nitrates and nitrites are absorbed into the bloodstream via the intestines. Nitrates and nitrites in the bloodstream also originate from systemic NO production systems).

The nitrate-nitrite-NO pathway, also known as the nitrate-nitrite-NO cycle, is a complex biological cycle referred to as the entero-salivary nitrate cycle (41, 42). After ingesting nitrate-rich foods, such as beetroot, lettuce, and spinach, the anion is rapidly absorbed from the upper GI tract, and around 75% of the nitrate is eliminated in urine within 24 hours (43, 44). The predominant portion of the residual nitrate is actively taken up by the salivary glands and secreted into the oral cavity. Symbiotic anaerobic bacteria resident on the dorsal surface of the tongue reduce nitrate to nitrite with the enzyme nitrate reductase. Salivary nitrite is subsequently swallowed and readily protonated to nitrous acid by the stomach’s acidic conditions, wherein it further degrades NO and other bioactive nitrogen oxides. Part of the salivary nitrite is taken up systemically without undergoing the reduction to NO within the stomach. Once absorbed, nitrite may be reduced to NO by various proteins and enzymes present in tissues and blood, such as hemoglobin, molybdenum enzymes, and mitochondrial enzymes (41, 45). (**Figure 1**).

Preclinical evidence indicates that generation of NO through these processes is significantly enhanced under hypoxia and acidosis, maintaining NO generation despite impaired activity of oxygen-dependent NOS enzymes. During physiological and pathological hypoxia, nitrite reduction to NO and NO-modified proteins have been implicated in physiological hypoxic signaling, vasodilation (11, 12), reduction of blood pressure, cellular regulation of respiration, and cellular protective effects against ischemia/reperfusion injury.

### 3.1 Factors that influence

#### 3.1.1 Sources

Human nitrate consumption has two primary sources: NO oxidation and direct intake from external sources (dietary and environmental) (46). Vegetables and drinking water are the primary dietary sources of nitrate (45, 47).

NO can be considered exogenous or endogenous, depending on where it is produced in the body. Exogenous NO is produced by reducing nitrates that occur in food and drinking water. This decrease is facilitated by facultative anaerobic bacteria found in the oral cavity, such as Actinomyces, Staphylococcus epidermidis, and Leuconostoc, which directly reduce nitrates to nitrites. The acidic conditions in the stomach provide a medium in which the reduction of nitrite to nitrous acid is possible, which spontaneously breaks down into NO and several other nitrogen oxides of biological significance (48). Endogenous NO production is catalyzed by NOS. In humans, the enzyme exists in three forms: neuronal NOS (nNOS), inducible NOS (iNOS), and endothelial NOS (eNOS) (49). NO from eNOS is vitally involved in normal endothelial cells functioning and CBF regulation (50, 51). The expression of nNOS in both the periphery and the center is crucial for memory and learning, as nNOS-derived NO regulates synaptic plasticity —a fundamental mechanism underlying both learning and memory (52, 53). Under healthy physiological conditions, inducible iNOS is minimally expressed but is inducible by a wide range of cytokines. Upon immune or microbial challenge, sustained iNOS expression leads to the production of large amounts of NO, a major mediator of inflammatory responses (54–56).

#### 3.1.2 Enzymatic pathways

Multiple enzymes are responsible for the reduction of nitrite to NO and other biologically active nitrogen species (57, 58). Some of them include those implicated in the bioactivation of nitrite, such as hemoglobin, myoglobin (59), neuromelanin (60), xanthine oxidoreductase (57, 61), aldehyde oxidase, carbonic anhydrase (62), eNOS, and mitochondrial enzymes (63).

#### 3.1.3 Redox microenvironment and regulatory factors

The activity of the nitrate-nitrite-NO pathway is not solely dependent on enzyme presence but is profoundly influenced by the local redox state. NADPH oxidase (NOX) and xanthine oxidoreductase (XOR) are key enzymes responsible for generating ROS. They deplete NO and reduce its bioavailability by producing superoxide anions (O_2_^-^), which react with NO to form peroxynitrite (ONOO^-^) (64). Interestingly, nitrates and nitrites themselves can feedback-regulate the activity of these oxidative enzymes. Studies have shown that dietary nitrate supplementation downregulates NOX activity in the vasculature and kidneys while simultaneously shifting the functional direction of XOR from ROS production to NO generation, thereby synergistically restoring redox balance (64, 65).

#### 3.1.4 Microbiota and dietary synergistic factors

Oral commensal bacteria constitute the primary step in nitrate activation, converting nitrate to nitrite via nitrate reductases. The use of antibacterial mouthwash can significantly impair this process, thereby affecting systemic NO bioavailability (66). Furthermore, the acidic environment of the stomach facilitates the non-enzymatic reduction of nitrite to NO, which contributes to gastric mucosal defense. The gut microbiota may also participate in the further metabolism of nitrates and nitrites (67). It is noteworthy that the dietary source of nitrate is critically important: nitrate derived from vegetables is often accompanied by vitamin C and polyphenols. These compounds enhance the efficiency of nitrite reduction to NO while simultaneously inhibiting the formation of potentially harmful N-nitrosamines (68).

The nitrate-nitrite-NO pathway is implicated in numerous diseases, particularly numerous neurologic disorders. This paper aims to discuss its implications in ischemic stroke.

## 4 Implications of the nitrate-nitrite-NO pathway for neuroinflammation and cognitive impairment following ischemic stroke

### 4.1 The role of neuroinflammation in ischemic stroke

**Figure 2:**
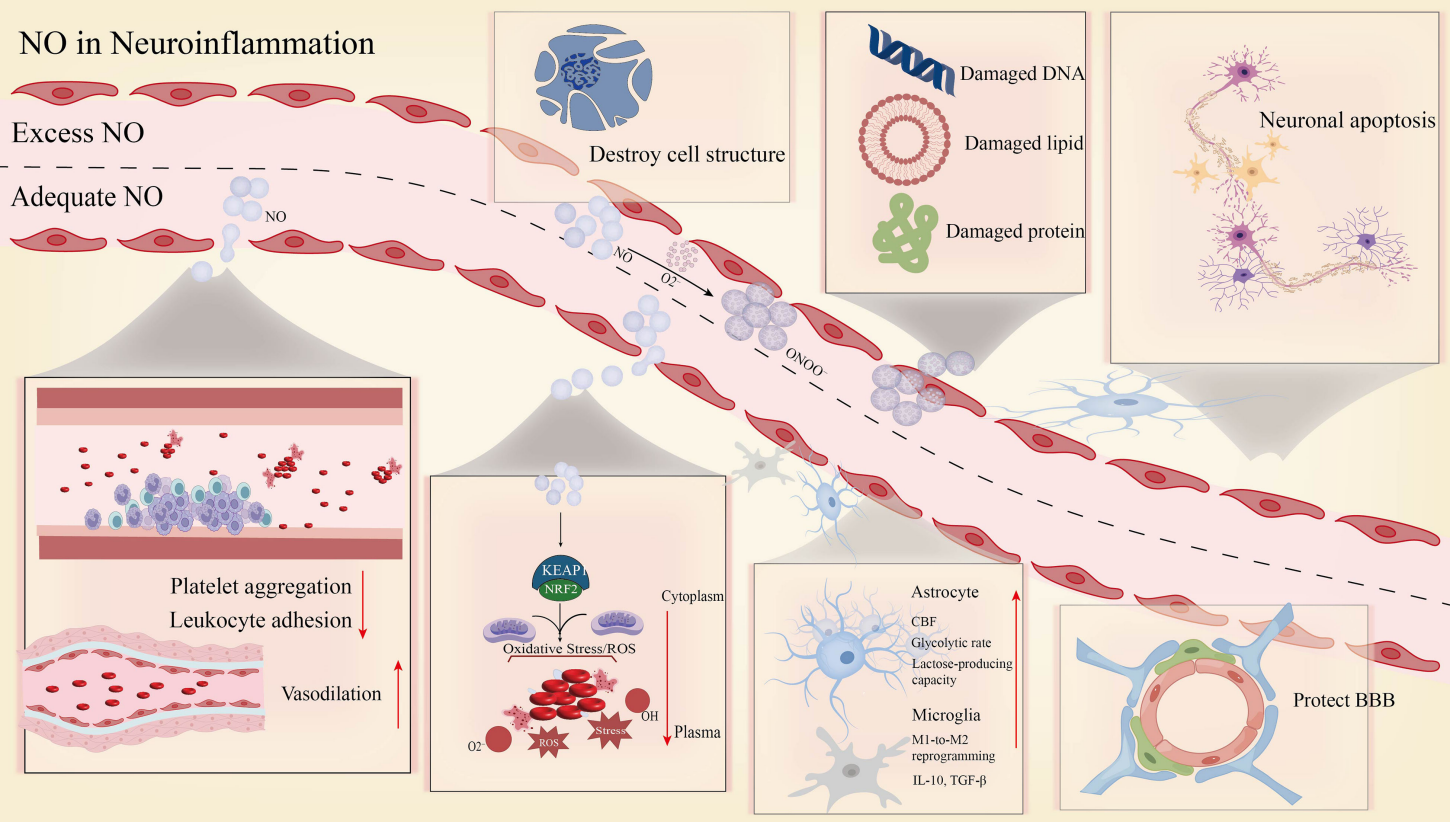
The role of exogenous NO in neuroinflammation. (Excessive NO disrupts cellular structures, leading to DNA, lipid, and protein damage alongside neuronal apoptosis. In contrast, moderate levels of NO exert complex dual effects through regulating relevant pathways. These include inhibiting platelet aggregation and leukocyte adhesion, promoting vasodilation, counteracting oxidative stress (e.g., via the KEAP1-Nrf2 pathway), influencing astrocyte and microglial function, and protecting the BBB).

NO is a pleiotropic messenger molecule and plays an essential role in numerous physiological processes, including the regulation of cellular homeostasis and neural, endothelial, and immune functions. NO performs two different tasks in neuroinflammation. It has been demonstrated in experimental models that the genetic induction of vascular NO increase provides robust neuroprotection after ischemic stroke (69, 70). However, overproduction of NO also causes toxic effects such as systemic inflammatory responses and neurotoxicity (**Figure 2**; **Table 1**).

**Table 1:** The dual role of nitric oxide in neuroinflammation following ischemic stroke.

Phase	NO source	Key mechanisms	Net biological effect
Ischemic Early Phase / Early Reperfusion (Anti-inflammatory/Neuroprotective)	➣ Nitrate–nitrite–NO pathway (preferentially activated under hypoxia) ➣Endothelial nitric oxide synthase (eNOS)	➣ Inhibits platelet aggregation and leukocyte adhesion (ICAM-1, VCAM-1, P-selectin) ➣ Scavenges reactive oxygen species; activates Nrf2-mediated antioxidant defense ➣ Promotes microglial/macrophage polarization toward M2 anti-inflammatory phenotype ➣ Enhances astrocytic glycolysis and lactate production, alleviating energy stress ➣ Induces microvascular dilation, improving perfusion in ischemic penumbra	Anti-inflammatory, Neuroprotective (Suppresses inflammation initiation, attenuates oxidative stress, preserves blood–brain barrier integrity)
Late Reperfusion Phase (Pro-inflammatory/Neurotoxic)	Inducible nitric oxide synthase (iNOS) was massively upregulated NO reacts with superoxide (O_2_^-^) to form peroxynitrite (ONOO^-^)	➣ Excessive NO and O_2_^-^ generate ONOO^-^, leading to protein nitration, lipid peroxidation, and DNA damage ➣ Activates pro-inflammatory signaling cascades (e.g., NF-κB), amplifying neuroinflammation ➣ Causes mitochondrial dysfunction and triggers neuronal apoptosis/necrosis ➣ Indirectly promotes excitotoxicity (via NMDAR modulation and increased glutamate release)	Pro-inflammatory, Neurotoxic (Exacerbates oxidative stress, disrupts cellular structures, propagates secondary injury)

#### 4.1.1 Anti-inflammatory and neuroprotective effects (early ischemia/early reperfusion)

NO initially inhibits platelet activation and aggregation, reducing the formation of microthrombi. Even more significantly, it prevents endothelial cell expression of adhesion molecules (e.g., ICAM-1, VCAM-1, P-selectin), thereby reducing infiltration of inflammatory cells (neutrophils, monocytes) from the circulation into ischemic brain tissue—a pivotal step in dampening neuroinflammatory initiation (71). Second, NO itself possesses free radical scavenging capacity (though it might also be implicated in ONOO^-^ production) (72) and indirectly enhances endogenous antioxidant defense by activating pathways such as Nrf2 (73), thereby limiting oxidative stress-induced neuronal damage—a major contributor to neuroinflammation. In addition, experiments have shown that optimal concentrations of NO possess the capacity to reverse polarization of microglia and infiltrating macrophages from pro-inflammatory (M1-like) to anti-inflammatory (M2-like) phenotype (74, 75). This change correlates with reduced levels of pro-inflammatory cytokines, such as TNF-α, IL-1β, and IL-6 (76, 77), and with elevated secretion of anti-inflammatory mediators, such as IL-10 and TGF-β.

In summary, NO averts neuroinflammation and protects the body through diverse mechanisms, including inhibiting platelet aggregation and leukocyte adhesion, opposing oxidative stress, and modulating microglial/macrophage polarization.

At the same time, NO plays a vital role in vascular relaxation (78). NO synthesized by this pathway enhances microcirculatory perfusion in penumbral ischemia (79), increasing flow to rescue neurons on the brink of death. Perfusion itself may be anti-inflammatory. Astrocytes govern CBF during hypoxia. Under reduced oxygen delivery, astrocytes have enhanced expression of sulfite oxidase, a mitochondrial molybdenum cofactor-containing enzyme. This enzyme enables nitrite reduction to produce NO, a potent vasodilator, thereby mediating parenchymal cerebral vasodilation and regulating CBF during hypoxia (80). In addition, NO-treated astrocytes exhibit a higher rate of glycolysis and an enhanced capacity for lactate generation (81), which significantly alleviates hypoxic energy stress. Furthermore, lactate is an intra-aneurysmal signaling molecule in the brain, where it modulates a wide range of essential functions, from neurovascular coupling (NVC) to metabolic homeostasis (82, 83).

#### 4.1.2 Pro-inflammatory and neurotoxic effects (late perfusion phase)

During late perfusion, when oxygenation is restored, excess production of NO via the NOS pathway (particularly following extensive induction of inducible iNOS) and interaction between NO of the NO_3_^-^-NO_2_^-^-NO pathway and simultaneously generated O_2_^-^ leads to excess generation of ONOO^-^ (84). ONOO^-^ is a highly potent oxidant and nitrosylating compound. Under pathological conditions, the formation of ONOO^-^ and oxygen-free radicals may overwhelm cellular antioxidant defenses, directly oxidizing lipids, proteins (especially critical enzymes), and DNA. Nitrated proteins (e.g., mitochondrial and neurofilament proteins) interfere with cellular structure and function (85) while concurrently triggering pro-inflammatory signal transduction cascades, such as NF-κB. This, in turn, further amplifies inflammatory responses, leading to mitochondrial dysfunction and neuronal apoptosis/necrosis (86). Excess NO also causes renal tissue damage (87), such as diabetic nephropathy and septic nephropathy. Furthermore, increased NO levels can indirectly contribute to excitotoxicity by modulating NMDA receptor function or by increasing glutamate release (88).

### 4.2 Cognitive impairment after stroke

**Figure 3:**
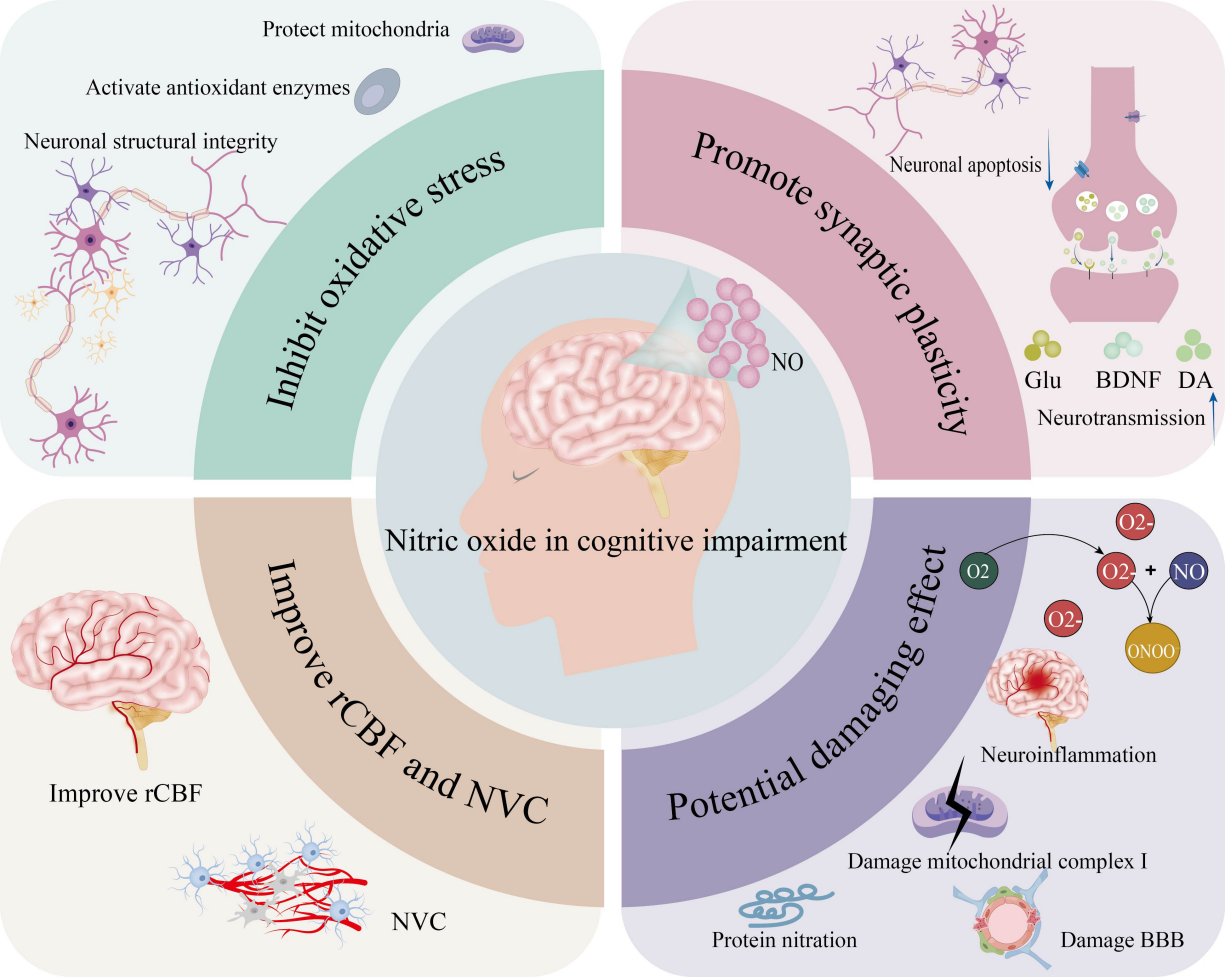
The role of exogenous NO in cognitive impairment. (On the one hand, it can suppress oxidative stress (protecting mitochondria, activating antioxidant enzymes, and maintaining neuronal structural integrity), promote synaptic plasticity (participating in neurotransmitter transmission and influencing neuronal apoptosis), and improve rCBF and NVC; On the other hand, it also possesses potential deleterious effects, including the potential to induce neuroinflammation, damage mitochondrial complex I, induce protein nitration, and compromise the BBB).

Pathophysiology of cognitive impairment following ischemic stroke involves damage to the cortex and hippocampus due to direct ischemia or secondary degeneration, initiating chronic neuroinflammation and blood-brain barrier breakdown, and ultimately leading to synaptic loss and neuron death, and also white matter injury (38, 89). The subsequent discussion will discuss the role of exogenous NO in post-ischemic cognitive impairment, primarily through its modulation of synaptic plasticity, suppression of oxidative stress, and augmentation of CBF perfusion and NVC. (**Figure 3**). **Table 2**.

**Table 2:** Nitric oxide-mediated mechanisms in improving post-stroke cognitive impairment.

Mechanism category	NO-mediated mechanisms	Functional improvement in cognition
Synaptic Plasticity Regulation	➣ Acts as a gaseous neurotransmitter, modulating synaptic transmission ➣ Activates CREB pathway via cGMP/cAMP signaling cascades, directly regulating synaptic plasticity and memory consolidation ➣ Influences neurotransmitter release and metabolism of glutamate, BDNF, and dopamine	Enhances learning and memory; restores synaptic function and neuronal connectivity
Cerebral Blood Flow Enhancement	➣ Activates nNOS to produce NO, stimulating sGC ➣Increases intracellular cGMP levels, inducing vascular smooth muscle relaxation ➣ Improves microcirculation in ischemic regions via the nitrate-nitrite- ➣ NO pathway under hypoxic conditions	Preserves energy supply to cognitive areas; supports neuronal survival and function
Neurovascular Coupling (NVC)	➣ NO from hippocampal neurons (via NMDA receptor-activated NOS) is essential for neurovascular control ➣ Maintains proper hemodynamic responses to neuronal activation ➣ Nitrate supplementation restores NO bioavailability, enhancing NVC	Preserves functional connectivity between brain regions; supports cognitive processing
Oxidative Stress Inhibition	➣ Induces antioxidant enzymes ➣ Protects mitochondrial function ➣ Reduces neuronal apoptosis ➣ Inhibits neuronal degeneration and synaptic protein dysfunction caused by oxidative damage Attenuates free radical damage	Maintains neuronal structural and functional integrity; prevents oxidative stress-induced cognitive decline

#### 4.2.1 Modulating synaptic plasticity

The characteristics of VD are chronic progressive cognitive deterioration, multifarious manifestations, and unpredictable course (50, 90–92). Its pathogenesis is defined by the impairment of synapton-synaptic neurons, endothelial cells, and microglia, along with neuroinflammation and autophagy (93–95). It is widely accepted that cerebral hypoperfusion is the most important cause of VD (96, 97). This process also leads to a cascade of synaptic impairment and neuronal degeneration or loss, ultimately resulting in cognitive impairment (94). Synaptic impairment caused by vascular injury is no doubt an early event in the pathogenesis of VD (98, 99). Synaptic plasticity refers to the activity-dependent change in existing synapses. Changes in number, morphology, and transmission effectiveness are fundamental neurobiological mechanisms underlying cognitive functions such as learning and memory (100, 101). NO, a critical gaseous neurotransmitter, conveys intercellular communication and modulates synaptic transmission in the CNS. Therapeutically, NO supplementation in NO-deficient states rescues synaptic plasticity, underscoring its therapeutic potential. Mechanistically, NO does so via the cGMP/cAMP signaling cascade, which feeds into the cAMP Response Element-Binding Protein pathway and directly regulates synaptic plasticity and memory consolidation (102, 103). Previous research has also demonstrated (104–106) that NO, due to its role in regulating synaptic plasticity, is a potential therapeutic target in VD. In VD, such complexity in NO generation regulation is evident.

Individual activities of solitary NOS isoforms establish kinetic features of CCH-induced alterations. This highlights their concerted action in precisely regulating NO production and its effects, which lead to oscillations in NO levels and profiles in brain tissue (97, 107). At the initial phase of stroke, the sudden decrease in CBF causes an acute energy crisis in ischemic brain tissues, triggered by a deficiency in glucose and oxygen. Concurrently, Ca²^+^ overload and membrane depolarization increase excitotoxic glutamate release, suppress eNOS activity, reduce NO secretion, and impair its availability (108). Another mechanism is also possible: post-stroke ROS oxidizing tetrahydrobiopterin (BH_4_, precursor for NO synthesis) to BH_2_, thereby reducing NO formation and suppressing eNOS activity (109). ROS-derived O_2_^-^ possesses a high capacity to be converted to ONOO^-^, increasing utilization of NO and suppressing downstream NO signaling. The nitrate-nitrite-NO pathway avoids NO release in acidic and/or hypoxic environments, thereby compensating for oxygen deficiency and delivering NO (32, 110).

A recent study demonstrated that a botanical mixture (BM) composed of fermented garlic extract, fermented Lactuca sativa (glucose-ethanol extract), and fermented Phaseolus angularis extract, supplemented with nitrite (2000 ppm) as a source of NO metabolites, ameliorates cognitive impairment anneural plasticity (111). Meanwhile, BM reduced neuronal apoptosis, GFAP expression, and oxidative stress, while increasing albumin and BDNF expression. The neuroprotective effect of BM, demonstrated by its ability to prevent cognitive impairment and promote neuroplasticity, indicates its therapeutic potential for VD treatment.

In summary, NO indirectly promotes neural signaling and regulates synaptic plasticity by influencing neurotransmitter release and the metabolism of glutamate, BDNF, and dopamine.

#### 4.2.2 Facilitating CBF perfusion and NVC

NVC, the spatiotemporally regulated interaction between CBF and neuronal activity, is a basic mechanism necessary for maintaining normal brain function (112). NVC dysfunction leads to reduced connectivity among different brain regions, thereby impairing cognitive function. Recent studies have also indicated (113) that NVC is a theoretical basis for age-related cognitive impairment. It preserves cognitive function by controlling the timely delivery of energy and oxygen to active areas of the brain, a process required for maximum neural computation. NO production by hippocampal neurons via NMDA receptor-activated NOS has been identified as a crucial mechanism in NVC, necessary for generating proper neurovascular responses. NO-mediated CBF responses, when dysregulated due to disruption of this pathway, cause neuronal activation that underlies cognitive impairment (114). Reduced NO bioavailability is associated with ineffective signaling that promotes a hemodynamic response to neuronal stimulation, potentially through increased clearance (e.g., by O_2_^-^) or limited NO generation. Hence, enhancing NO bioavailability to counteract neurovascular dysfunction is crucial. It has been reported (115) that acute infusion of nitrite enhances CBF in rats and reconstitutes hemodynamic responses to exercise in conditions of NOS inhibition. Simultaneously, nitrates initiate a signal cascade by activating nNOS, which generates NO. This significant signaling molecule acts as a neurotransmitter, stimulating effector cells and triggering NO-sensitive sGC (116, 117). The principal activity of this sGC activation is to increase intracellular cGMP levels, thereby relaxing vascular smooth muscle cells (118), ultimately supporting standard NVC function (119). Fundamentally, Nitrite (NO_2_^-^) is recognized as a central physiological regulator of hypoxia signaling in mammals, maintaining cardiovascular health through NO-dependent mechanisms and supporting normal arterial function in aged individuals. Moreover, some studies indicate that the intake of nitrate-rich foods, such as beetroot juice, can modulate human CBF responses to prefrontal cortical tasks assessed by near-infrared spectroscopy and enhance performance in a continuous 3-second subtraction task (120). As a result, supplementation with NO_3_^-^ enhances NO bioavailability, enhances NVC, protects against energy supply to cognition-related brain regions, and promotes synaptic transmission and neural network function.

Also, in all age groups, increased systemic inflammation and oxidative stress have been established as the leading risk factors for dementia and cognitive impairment. In the brain, increased oxidative stress alters normal cellular processes and destroys neuronal structure and functional integrity (121–123). NO reduces oxidative stress damage through several mechanisms in the body (72). NO suppresses free radicals and enhances cellular resistance to oxidative stress by inducing antioxidant enzymes, protecting mitochondrial function, reducing neuronal apoptosis, and inhibiting neuronal degeneration and synaptic protein dysfunction caused by oxidative damage. Additionally, elevated NO levels are reported to be neurotoxic (124, 125), with biological activity being concentration-dependent.

Excess NO injury manifests as nitration of α-synuclein (126). Nitrating of tyrosine residues within proteins is increasingly implicated in neurodegenerative disorders as a central pathogenic process (127), with most NO-mediated neurodegenerative disorders found to be nitration-induced. NO overaccumulates and destroys mitochondrial complex I, leading to energy depletion in neurons (128), amplifying the vicious cycle of neuroinflammation and accelerating cognitive decline. Persistent disruption of the blood-brain barrier allows peripheral inflammatory cells and fibrinogen to migrate into the brain parenchyma, triggering glial cell activation and white matter damage.

## 5 Therapeutic strategies

### 5.1 Improving CBF and vascular function

One of the earliest physiological effects of NO demonstrated is the mediation of vasodilation in the cardiovascular system. NO stimulates guanylate cyclase (GC), raises cyclic guanosine monophosphate (cGMP), causes vascular smooth muscle relaxation, and raises CBF (129, 130). NO donor medications, such as nitroglycerin (131) and sodium nitrite, enhance NO targeting in the brain when paired with nanocarriers (132, 133), such as liposomes and polymeric nanoparticles. This approach promotes the instantaneous release of NO, cerebral vasodilation, reduces systemic side effects, and increases blood supply to ischemic regions.

### 5.2 Antagonism of neuroinflammatory response

NO modulates microglial activity by inhibiting the release of proinflammatory mediators, reducing oxidative stress, and inducing the expression of anti-inflammatory factors (28). Low-dose NO donors can prevent oxidative stress induced by high doses but selectively block microglial overactivation. Its administration, combined with nonsteroidal anti-inflammatory drugs (NSAIDs) (112) or immunomodulators, synergistically reduces neuroinflammation. Exogenous NO inhibits NF-κB translocation to the nucleus by modifying its critical cysteine residues, thereby preventing the transcription of proinflammatory cytokines (e.g., TNF-α, IL-1β) (134, 135). Moreover, low concentrations of NO scavenge O_2_^-^ directly, reduce ONOO^-^ production, and reduce oxidative damage.

### 5.3 Neuroprotection and repair

NO neuroprotects and facilitates axonal regeneration by preventing apoptosis (e.g., Caspase-3 inhibition) and promoting neurotrophic factors (e.g., BDNF). Thus, synergy between NO donors and neurotrophic factors, such as co-administration of NO donors with BDNF, enhances neuroprotective activity. BDNF enhances synaptic plasticity and memory by inhibiting autophagy under conditions of nutritional stress (136). Concurrently, NO imparts neuroprotection through the significant upregulation of BDNF and the inhibition of autophagy, which are potential contributory mechanisms. Statins, widely used in the treatment of stroke and atherosclerosis, such as lovastatin, reduce oxidative stress, prevent apoptosis, and provide neurovascular protection (137). Despite having apparently therapeutic effects, these medications still require robust clinical evidence.

### 5.4 Emerging therapeutic approaches

In the past few years, the development of new technologies for treating diseases has been a leading trend. Medicine’s short half-life (approximately 5 seconds) implies that stable and controllable delivery systems should be designed (138). Experiments have demonstrated that light-gated or pH-sensitive nanoparticles may offer spatially controlled NO release at the site of the lesion (139). Moreover, intranasal delivery can circumvent the BBB and deliver NO directly into the brain, thereby increasing bioavailability (140). Brain drug delivery and targeted drug release, utilizing the distinct properties of the cerebral vasculature, can significantly enhance drug targeting efficacy and therapeutic efficiency. In addition, gene therapy—in the form of viral vector delivery of the iNOS gene (rAAV/iNOS) can maintain long-term upregulation of endogenous NO production, improving long-term prognosis (91, 132, 141–145). MiR-501-3p has also been proposed as a candidate for treating VCI, with mouse model studies indicating its ability to slow disease progression (89, 146). Meanwhile, PANO gel that releases exogenous NO has emerged as a novel therapeutic approach for metabolic disease and cognitive dysfunction (147). These new technologies work through different mechanisms to treat cognitive dysfunction.

### 5.5 Clinical applications

NO therapy in neurology remains largely investigational, primarily targeting CBF enhancement and neuroprotection. In ischemic stroke, NO donors (e.g., nitrates) induce vasodilation, increasing perfusion to ischemic regions and reducing hypoxic injury, though this approach remains in the preclinical and early clinical stages. Exogenous NO also alleviates cerebral vasospasm following subarachnoid hemorrhage by promoting vascular smooth muscle relaxation (148). Recent large-scale trials have revealed complex, time-dependent effects of NO-based therapies. The RIGHT-2 trial (n=1,149) demonstrated that prehospital transdermal glyceryl trinitrate (GTN) lowered blood pressure but failed to improve functional outcomes and may have worsened intracerebral hemorrhage (149). A 2025 meta-analysis (3,547 patients) found no significant benefit in mortality or functional recovery. Expert consensus suggests GTN is safe within 2–6 hours post-stroke but should be avoided ultra-acutely (<2 hours) (150). Additionally, some studies suggest that exogenous NO may alleviate neuropathic pain by modulating neurotransmitter release (e.g., inhibiting norepinephrine release), though these findings require validation in large-scale clinical trials and are not currently incorporated into routine clinical practice (151–153).

## 6 Conclusion and outlook

Ischemic stroke leads to severe consequences, neuroinflammation, and VCI. Thus, prevention and reduction of stroke- and infarction-induced cerebral damage have been the primary focus of therapeutic research. This review article discusses neuroinflammation and cognitive impairment caused by ischemic stroke, relief and repair of such damage by exogenous NO, and proposes some therapeutic options. Clinical evidence for NO therapy has primarily focused on vasodilation in the past decade, and its potential applications have been limited to cerebrovascular disorders. Its use in the discipline of neurology has yet to gain acceptance. However, numerous medications have demonstrated interesting neuroprotective potential in preclinical models.
